# Supplementary material for: Molecular basis of force-from-lipids gating in the mechanosensitive channel MscS
Source: eLife. 2019 Dec 27;8:e50486. doi: 10.7554/eLife.50486 (PMC7299334; doi:10.7554/eLife.50486)
Supplement: Supplementary file 1 [file elife-50486-supp1.odt]

Table S1. Cryo-EM data collection, refinement and validation statistics 

|  | MscS ND His | MscS ND No His | MscS DDM |
| --- | --- | --- | --- |
| Number of Movies | 4403 | 2110 | 1328 |
| Magnification | 130k | 130k | 130k |
| Dosage e- /Å2 | 50 | 50 | 50 |
| Pixel size (Å) | 0.532 | 0.532 | 0.532 |
| Voltage (kV) | 300 | 300 | 300 |
| Defocus Range (µm) | 1-2.5 | 1-2.5 | 1-2.5 |
| Selected particles | 1064935 | 104765 | 72501 |
| Final number of Particles | 372870 | 15071 | 50782 |
| Symmetry imposed | C7 | C7 | C7 |
| Map resolution (Å) (0.143) | 3.1 | 4.1 | 3.4 |
| Model refinement |  |  |  |
| Model composition |  |  |  |
| Non-hydrogen atoms | 15379 | 14903 | 14217 |
| Protein residues | 1960 | 1960 | 1862 |
| Ligands | 14 |  |  |
| B factors (Å2) |  |  |  |
| Protein | 113.55 | 273.32 | 148.44 |
| Ligand | 144.32 |  |  |
| Validation |  |  |  |
| MolProbity score | 1.67 | 2.31 | 1.49 |
| Clashscore | 7.99 | 23.73 | 6.59 |
| Poor rotamers (%) | 0 | 0.31 | 0 |
| Ramachandran plot |  |  |  |
| Favored (%) | 96.35 | 93.17 | 97.35 |
| Allowed (%) | 3.65 | 6.83 | 2.65 |
| Outliers (%) | 0 | 0 | 0 |

Table S2. Various all-atom molecular dynamics models used in this study.

| Model # | Description | Number of Atoms | Number of lipids | Box size | Equilibration time (ns) |
| --- | --- | --- | --- | --- | --- |
| 1 | No Lipids | 415319 | 685 | 160x160x171 | 60 |
| 2 | Hook Lipid | 411110 | 677 | 160x160x171 | 60 |
| 3 | Both lipids | 416294 | 684 | 160x160x171 | 60 |
| 4 | CHARMM GUI prediction | 412629 | 680 | 160x160x171 | 60 |
| 5 | Highest bilayer position | 423300 | 695 | 160x160x171 | 60 |
